# Predictors of Feeling of Threat Caused by COVID-19 Pandemic, the Distinctive Effects of Automatic vs. Reflective Emotions

**DOI:** 10.3390/ijerph20075231

**Published:** 2023-03-23

**Authors:** Maciej Pastwa, Kamil K. Imbir, Adrianna Wielgopolan, Ernest Adach

**Affiliations:** Faculty of Psychology, University of Warsaw, 00-183 Warszawa, Poland

**Keywords:** COVID-19, feeling of threat, negative emotions, automatic emotions, reflective emotions

## Abstract

The worldwide pandemic that started in December 2019 was a cause of a great rise in the feeling of threat in society. A feeling of threat and distress can be influenced by the span of emotions experienced by a person, and as it is rather clear, that the situation of pandemic evokes negative emotions, they can range from fear to depression, to even disgust. In this study, we wanted to verify the influence of the negative emotions of automatic origin, related to the well-being and homeostasis of the organism and the negative emotions of reflective origin, which are related to social constructs, on the feeling of threat caused by the pandemic outbreak. We expected automatic emotions to have a greater influence on the feeling of threat. We used an online questionnaire to measure the intensity of negative emotions and the feeling of threat among Polish participants in the time of the early outbreak of the pandemic (March–April 2020). Regression analyses were used to identify the predictors of the feeling of threat. The results show the distinctive effect of automatic and reflective groups of emotions. While automatic emotions always increased the feeling of threat, the reflective emotions suppressed the distress, especially in the group of middle-aged and elderly participants. As reflective emotions are developing in the process of socialization, the observed results could suggest, that young people do not process the situation of the pandemic in reflective categories, which leaves them more worried about the situation. We suggest, that promoting reflective thinking can be helpful in interventions in the cases of anxiety caused by the pandemic, as well as in social communication regarding the topic of the pandemic.

## 1. Introduction

The outbreak of the coronavirus pandemic at the end of 2019 was not only a threat to the health of people all over the world but also a cause of great stress and anxiety when the virus was detected. It was immediately outlined, that the worldwide pandemic will be a factor of a great rise in anxiety levels in society and that a long time will be needed to recover from the psychological consequences of the pandemic [1,2,3,4,5].

The prognosis was proven to be right by the early studies of stress and anxiety related to COVID-19. In China, right after the outbreak, the symptoms of those negative feelings became more frequent among the population [6,7,8,9,10,11,12]. This raise in stress and anxiety symptoms also could be observed in other countries seriously damaged by the virus in the early stage, namely Italy and the USA [13,14], but also in countries less damaged by the pandemic’s first outbreak, like India or Singapore [15,16,17]. In Poland, where the presented study was conducted, there was also an immediate raise in the level of experienced anxiety, the feeling of threat and negative emotions [18,19,20,21]. The aim of the presented study was to identify emotional, cognitive and demographic factors that could increase the COVID-related feeling of threat, or be protective against it.

### 1.1. Automatic and Reflective Emotions

The outburst of the pandemic evoked a strong emotional reaction, consisting almost entirely of negative feelings. The span of experienced emotions, which could be very wide even among the negative emotions themselves, may influence the anxiety felt by a person. As a response to the pandemic situation, people may experience different emotional states, being—in a way—an informative barometer about their surroundings [22,23] and giving them clues on how to appropriately appraise and interpret the situation [24]. Even extremely negative emotions may be beneficial in a dangerous situation, as they give a warning message that there is something wrong and a quick reaction is needed [25]. Negative emotional states may also elicit a more systematic, reflective processing of information, therefore causing an individual to be more cautious and attentive to deal with a situation better [24]. Costs of hedonistic regulation and disregarding the negative effect caused by some threats may be catastrophic for the well-being of an individual.

The diversity of human emotions can be described in several ways [26,27,28], including the discrete emotions approach (distinguishing for example happiness or anger) as well as the dimensional approach (focusing on dimensions such as valence or arousal). Among different approaches to emotions, the duality of mind seems to be the most promising in explaining the diversity of emotional states experienced in everyday situations [26,29,30,31]. This model postulates that the so-called origin (automatic vs reflective) of emotional reaction is the crucial factor allowing for an understanding of the consequences of emotion [29]. The origin ascribes cognitive mechanisms responsible for emotion formation. The automatic origin represents an immediate reaction to the stimulus, that does not require elaborated cognition to appear [32,33]. Such emotions are primary in a developmental sense and each time appear earlier than any other emotions. For example, Damasio [32] postulated biological value as the automatic criterion of evaluation that gives an organism a chance to get to know what helps survive without reasoning (sweet meal is caloric, thus triggering positive feelings e.g., joy). Such emotions are automated and can drive simplified, heuristic cognition [29,34,35]). 

Among automatic-originated emotional states, one may distinguish between: (1) automatic homeostatic emotions and (2) automatic aversive emotions. Automatic homeostatic emotions are related to internal homeostasis maintenance (e.g., fear of a predator, feeling of pain, relaxation after exhaustion), to the biological and psychological states, being an answer to some deprivation or gratification [26]. Automatic aversive emotions are related to the external aversive or hedonistic experiences, a regulator of behaviour towards some stimuli, based on the fact whether said stimuli is pleasant or not (e.g., aversive reaction to rotten food, but, on the contrary, approaching a warm object) [26,29].

The reflective origin represents an elaborated interpretation of the stimulus meaning that leads to the emotional reaction. Such a reaction has to be more delayed in time but is more plastic and subject-dependent. The emotional reaction comes from the interpretation of stimulus or the meaning of the situation in the context of some explicitly stated criteria of evaluation [26,31,36] that are verbalized and represented in a propositional code [35], therefore their acting in guiding emotional reactions is not as obvious as the influence of automatic originated emotions. Nevertheless, when present, reflective-originated emotions can provide completely different outcomes, such as promotion of the rational thinking, consideration or contemplation [29,37,38]. 

Among reflective-originated emotional states, one may distinguish between: (1) emotions related to the Self standards and (2) emotions not related to the Self. Self, in this context, may be understood as the representation of one’s identity, perceived on both a cognitive and affective level, playing role in motivation, constructing social identity, regulation of emotions and other processes, in many cases through the desired standards imposed by it [39]. Reflective emotions related to the Self standards are outcomes of cognitive appraisal, the process of comparing one’s situation to a certain situation desired by the perfect Self. Such an appraisal may result in, for example, pride in achievement or shame caused by a failure. Reflective emotions not related to Self-standards are also derived from a cognitive appraisal, but the points of comparison are outside factors like social norms or one’s view of the future. Sadness could be interpreted as a reflective emotion not related to the Self, as it is caused by the appraisal of one’s condition as bad due to factors that could not be easily changed, such as a loss or a disease. The distinction between these two groups of emotions is based on the focus point they motivate to fixate on—while the ones related to Self standards promote focusing on oneself as the reason for and the object of the emotional state, the ones not related to Self motivate to focus on the outside circumstances [26,29].

Reflective-originated emotions may play an important role in extreme situations. People tend to seek balance in emotional functioning, which is a healthy state, providing emotional comfort and well-being. However, during the pandemic, it is difficult to counterbalance negative emotions with positive ones, while the unhindered growth of negative emotions may lead to truly fatal outcomes [40,41]. As the spreading disease is a great factor generating negative emotions [8], people tend to seek positively valenced posts on social media and create posts in such a manner, especially regarding the emotions of compassion and hope [42]. A similar situation can be observed in the study conducted on people heavily touched by the pandemic—the caregivers, that took care of people suffering from COVID-19. As their initial reaction to the situation was highly negative, consisting of stress, anxiety and depression symptoms, as time passed, their feelings started to change. They started to find growth in this situation, experiencing compassion and gratefulness [43]. Among the general population on the other hand, we could see a decrease in the intensity of negative emotions as the pandemic lasted, which could mean that the span of experienced emotions changed, with more reflective-originated feelings counterbalancing the strong initial automatic reaction [20]. As reflective emotions are the ones bringing homeostasis in a situation of crisis, we can expect that, generally, the pattern of emotional functioning involving reflective emotions may be protective against negative outcomes of stress.

A study conducted by our research team brings some insight into the protective role of emotions in the pandemic situation [21]. In a comprehensive regression model, investigating the influence of different emotional categories on the experienced anxiety, we found that automatic negative emotions were increasing the anxiety, while reflective negative emotions had a protective role against it. The models have also shown differences between factors influencing anxiety in different age groups—while for young adults only the negative emotions were significant predictors of anxiety, in the group of older adults (above 40 years old) the positive emotions (both automatic and reflective) were protective against anxiety. 

The different patterns of emotional experiences in different age groups may be a more common phenomenon observed during the rise of the pandemic [18,21,44]. Considering different mechanisms of elicitation of automatic vs reflective emotional states, we have to deal with a developmental issue. In general, a system of evaluative standards should develop with age [26,29]. The older you are, the more experience you collect, and thus the more precise interpretations of what is desirable vs undesirable you can provide as the base for evaluation which leads to reflective emotions [35,36]. This wider range of emotional experiences may lead to better emotional well-being in general, which was also found to remain among the older age groups during the early outbreak of the COVID-19 pandemic [44].

Some studies suggest that young people may experience a lot of stress during the pandemic [9,18], while others show that the oldest people are the ones mostly suffering from distress and anxiety [10,18,45,46]. Firstly, the observed inconclusive results may be tied exactly to emotional functioning—as younger people may tend to feel more emotions with the automatic origin, which works fast and may be overwhelming, the older people may find themselves experiencing more emotions with the reflective origin. As the emotions with the reflective origin have a slower, more discrete influence on one’s mood and behaviour, the results regarding fear, distress and depression during the pandemic may depend on the time period of collecting the data—if it is late into the pandemic outbreak in a certain area, the reflective emotions may have influenced the feelings of citizens, especially among older groups. Secondly, the relation between age and distress may be modulated by following media and news, as there is a great difference in media consumption between age groups. While young people obtain information mostly from social media and websites, older people rather follow legacy news reports, like radio and television. Moreover, older people find it more difficult to sort out fake news, and that kind of news may lead directly to more stress and anxiety [47]. Taking into consideration changes in emotional functioning dependent on the age we expected the emotions to influence anxiety differently for participants of different ages. It is important to know, that it is a well-known fact now, that age is an important risk factor for severe COVID-19 [48], however, it was not common knowledge during the first outbreak of the pandemic.

### 1.2. Other Factors Influencing the Feeling of Threat

It is important to outline other factors, that may influence the feeling of threat regarding the coronavirus pandemic, as they should be included in a model verifying the predictors of a feeling of threat. First of all, studies show that having close contact with people, that were infected or suspected of infection, was a significant factor in increasing distress [6,11]. Therefore, we thought that in the presented study we should control this factor. Secondly, as many families suffer from financial loss during the outbreak of the pandemic [49], due to the change in their employment status or even lack of work, we thought that it would be accurate to control this factor in the presented study. This is supported by data from other studies, where having a full-time job or a steady source of income has been proven to be an important factor in preventing distress or anxiety [6,11,18].

There is evidence that women and men can function differently in the field of emotions. Women could be more vulnerable to anxiety than men, which could be a result of socialization processes, in which both men and women have to face specific expectations, including expression of anxiety and ways of how they will handle it [50]. Also, recent studies show the difference in emotional reaction to the pandemic between men and women—females are more endangered by distress and anxiety [9,14,19,51], however, some studies suggest, that the differences may be observed only among older adults [18].

In the face of the pandemic informing society about the spread of the disease and its fatality is crucial for the safety of the general public. If people have trust in the news source they follow and feel that they are getting enough information, it supports their well-being. Recent studies show that many people in fact do not believe the information provided by the administration and news reports, which is connected with higher distress and anxiety [11]. The lack of trust in news and media is also fueled by the news frequently contradicting each other, especially on social media, where information can be riddled with fake news or conspiracy theories—this phenomenon is called “infodemic” [52]. Misinformation may also lead to irrational behaviours (e.g., panic buying) and more extreme opinions on the actions taken by the administration [53]. However, governments all over the world had to provide new policies and restrictions in the face of the pandemic, not only communicating to the public about them, but also ensuring their execution. It is crucial that people believe that actions taken by the administration are right, as it allows them to submit to new restrictions and policies. Nevertheless, the relationship between trust in the government and distress caused by the virus seems complex—as the respect for authority increases the fear of self, the trust in actions taken by the authority may reduce fear about others [53]. The lack of trust in both news and the administration’s actions can be connected with the belief in conspiracy theories, as they bring easy explanations and are in line with the disbelief in mainstream information.

Studies show that people are more likely to believe in conspiracy theories because of specific emotions. For example, feeling uncertain caused by experiencing distress makes conspiracy beliefs stronger [54]. A similar tendency can be seen when people are anxious, as they are more likely to believe in conspiracy theories, as it helps them to deal with fear [55]. The same mechanism can be observed in the feeling of alienation and powerlessness [56]. Believing in conspiracy theories is mainly a result of the need for closure. People have a desire to obtain a straight answer to any question, leaving no space for confusion [57]. This is why, if in the case of pandemic people do not believe the media source and government, they may seek the easy explanation in conspiracy theories, which may be even amplified by distress and anxiety. We decided to measure the belief in such theories in the presented study, to check if it comes into relation with the feeling of threat caused by the pandemic.

### 1.3. Aim and Hypotheses

The aims of the study were: (1) Exploring the factors (emotional; demographic; conspiratorial thinking) increasing the feeling of threat caused by the coronavirus pandemic outbreak in Poland or reducing the feeling of threat; (2) Verifying the role of automatic and reflective originated emotions in shaping the magnitude of the feeling of threat during the outbreak of the pandemic.

We expected reflective-originated emotions to reduce the feeling of threat. Such a reductive role could be more intense in the older groups of participants, due to the developmental aspect of reflective-originated emotions—we wanted to explore how the emotions could influence anxiety in different age groups. This expectation is also supported by other studies conducted during the early stage of the COVID-19 pandemic in Poland, which showed different patterns of emotional functioning for different age groups [18,21]. Furthermore, we expected differences in the experienced anxiety between men and women, namely we expected women to declare higher distress. We also expected that people with a full-time job position would experience a smaller feeling of a threat than the ones without a job or working part-time. Regarding conspiracy theories we expected the high levels of belief in them to increase the feeling of threat.

## 2. Materials & Methods

### 2.1. Participants

The data was collected between 25 March 2020 and 12 April 2020—immediately after the state of the pandemic was declared by the Polish Government. 1128 people initially clicked the link to the study. We removed all participants that did not finish the whole study, and then we chose only the participants that finished in time differing maximally 2 standard deviations from the mean time of the completion (*M* = 556.27 s; *SD* = 812.25 s). We did this to exclude participants that fulfilled the survey in a few takes—as the study was conducted online, it was difficult to control the process of answering the questions. The final sample constituted 575 Polish internet users: 436 women (75.8% of the whole sample), 135 men (23.5%) and 4 others (0.7%). Participants were aged between 18 and 74 years old (*M* = 29.38; *SD* = 9.34). 6 participants declared they finished only primary education (1%), 148 finished secondary education (25.7%), 90 declared finishing post-secondary schools (not Universities; 15.7%), 119 finished University Bachelor’s degree (20.7%), 212 Master’s degree (36.9%). 151 participants declared living in a village or a small town (less than 50,000 citizens; 26.3%), and the rest of the participants lived in larger towns and cities. 248 participants declared having a full-time job (43.1%), while 276 declared having their payment reassured during the pandemic (48%)—these were two separate questions, one regarding the job, the other the payment. 5 participants stated that they have been diagnosed with COVID-19 (0.9%), and 25 of them declared that they knew someone infected (4.3%). Participants were informed that the questionnaire is anonymous, and they can stop answering the questionnaire at any moment. The design and consent procedure was approved by the ethical committee at the University of Warsaw, Faculty of Psychology.

As for Poland’s outline of the beginning of the pandemic, patient zero was documented on 4 March 2020. After that, the increase in people infected by coronavirus was slow, but steady, reaching 1051 people by 25 March 2020 (when we started data collection) and 6674 people by 12 April 2020 (we stopped gathering data at this point). However, in this time period, the Polish government decided to introduce a number of different restrictions: Polish borders were closed, universities and schools shifted to remote learning, and people could not gather in groups larger than 2 people other than family. Restaurants, bars, pubs, museums, galleries and shopping malls were also closed. Only the workplaces considered essential were open, which means mainly small shops, factories, gas stations, drug stores and healthcare services. The timing of data collection in our study allowed us to capture the very early response to the pandemic as an entirely novel situation in Poland.

### 2.2. Design 

The study involved a measure of the feeling of threat related to the COVID-19 pandemic, treated as a dependent variable. The independent variables were: emotions of different origins (automatic homeostatic; automatic aversive; reflective emotions related to Self standards; reflective emotions not related to Self standards—the exploration of the last group revealed two distinct factors within it: sadness-like and contempt-like emotions, see also Section 2.3.3. Emotions); conspiracy beliefs; demographic factors: gender, age, education, place of living or having a full-time job. 

The data were collected in an online questionnaire and stored in a digital database. We wanted to analyse the data in three stages: verifying the correlates and factors differentiating the intensity of feeling of threat; verifying the predictors of the feeling of threat in regression models; exploring the influence of interaction between emotional functioning and age on the feeling of threat.

### 2.3. Materials

The online questionnaire consisted of 4 blocks. First, we asked participants about the feeling of threat caused by the novel coronavirus in specific areas of life. We decided to ask about the feeling of threat first, to avoid the answers being influenced by other measures used in the study. Secondly, we measured conspiracy beliefs with the 15-item Generic Conspiracist Beliefs Scale [58]. The third block was a list of 20 emotions, the participants were asked to indicate how strongly do they feel the particular emotions during the pandemic situation. In the last block, the participants were asked about their anonymous particulars. A table with the summary of all used materials and validity statistics may be found in Supplementary Material.

#### 2.3.1. Feeling of Threat Caused by the Pandemic

The dependent variable in this study was the feeling of threat evoked by the pandemic. We used a set of three questions to measure this variable, the questions were asked in Polish language, presented in random order. The three questions we used were: As regards the current situation, to what extent do you feel physically threatened (aches, pains, illness)?; To what extent do you feel mentally threatened (concerns, anxiety, fear, panic)?; To what extent do you feel threatened by the dynamics of the pandemic (fear of contracting the virus)?. Answers were given on a scale from 1—“I definitely do not feel threatened” to 5—“I definitely feel threatened”. The scale achieved Cronbach’s *α* = 0.77. We have run a confirmatory factor analysis (PCA method with VARIMAX rotation) for this scale. The FA revealed only one significant factor (eigenvalue > 1), percentage of explained variance = 68.53%; *KMO* = 0.70; *χ*^2^(3) = 450.05, *p* < 0.001), with Factor Loading for the questions ranging from 0.82 to 0.85. As we wanted to measure feelings evoked in response to the novel situation of the pandemic, we created our own tool instead of adapting an existing one, but comparing Cronbach’s *α* with another similar scale [59] shows, that the data collected with the scale developed by our team can be reliable for conclusions.

#### 2.3.2. Conspiracy Beliefs

Conspiracy beliefs were measured with the 15-item Generic Conspiracist Beliefs Scale [58]. The GCB Scale measures individual differences in generic conspiracist ideation. The scale is practically useful, as it demonstrates internal reliability, content, criterion-related, convergent and discriminant validity, as well as good test-retest reliability [58]. The questionnaire of belief in conspiracy theories reached very high reliability in our sample (Cronbach’s *α* = 0.93). Respondents were asked to indicate the degree to which they believe each statement is likely to be true on the scale (1—Definitely not true, 5—Definitely true). Every 3 items were measuring different factors. Five factors were measured in this questionnaire, namely: Government Malfeasance (Cronbach’s *α* = 0.81), Malevolent Global (Cronbach’s *α* = 0.87), Extraterrestrial Cover-up (Cronbach’s *α* = 0.85), Personal Well-being (Cronbach’s *α* = 0.78) and Control of Information (Cronbach’s *α* = 0.73). The translation we used was not the one used in the Polish adaptation of the scale, however, it achieved comparable values of reliability statistics (Cronbach’s alpha for the Polish adaptation of the scales is 0.93, with alphas for subscales ranging from 0.70 to 0.88) [60].

#### 2.3.3. Emotions

Respondents were asked: Indicate to what extent you feel this emotion during the situation of the pandemic; the question was followed with a table with 20 emotions and scales next to them. They were indicating their answers on a continuous scale, without any numbers, where the left side meant “I feel the emotion to a small extent”, while the right side meant “I feel the emotion to a significant extent”. When participants opened the page with this part of the survey, the indicator of the decision was always in the middle of the scale for each emotion. The data was saved on a scale ranging from 1 to 100. The emotions were listed in randomized order for each participant. 

We measured 4 separate groups of emotions: automatic homeostatic; automatic aversive; reflective related to Self standards; reflective not related to Self standards; 5 emotions in each group. We decided to verify the a-priori set division of negative emotions. The group of automatic homeostatic emotions consisted of *Suffering*, *Helplessness*, *Frustration*, *Breakdown* and *Terror* and reached Cronbach’s *α* = 0.87. The group of automatic aversive emotions consisted of *Bitterness*, *Aversion*, *Disgust*, *Abhorrence* and *Repulsion* and reached Cronbach’s *α* = 0.86. The group of reflective emotions related to Self standards reached a lower Cronbach’s *α* = 0.78, thus we decided to run an exploratory factor analysis (PCA method with VARIMAX rotation) within this group of emotions. The FA revealed only one significant factor (eigenvalue > 1) within this group (Percentage of explained variance = 53.78%; *KMO* = 0.72; *χ*^2^(10) = 939.11, *p* < 0.001), it consisted of *Humiliation* (*Factor Loading* = 0.70), *Shame* (*FL* = 0.68), *Embarrassment* (*FL* = 0.67), *Disappointment* (*FL* = 0.80) and *Disillusionment* (*FL* = 0.80), Reflective emotions not related to Self standards also reached a lower Cronbach’s *α* = 0.75, exploratory FA (*KMO* = 0.66; *χ*^2^(10) = 1143.94, *p* < 0.001) revealed two significant factors within this group (Percentage of explained variance: Factor 1 = 46.54%, Factor 2 = 31.65%): sadness-like emotions, which were *Sadness* (*FLs*: Factor 1 = 0.88; Factor 2 = 0.02), *Sorrow* (Factor 1 = 0.93; Factor 2 = 0.09) and *Depression* (Factor 1 = 0.81; Factor 2 = 0.24), Cronbach’s *α* = 0.86, and contempt-like emotions, which were *Envy* (Factor 1 = 0.19; Factor 2 = 0.86) and *Contempt* (Factor 1 = 0.04; Factor 2 = 0.88), *r* = 0.55; *p* < 0.001.

### 2.4. Procedure

The study was conducted using the Qualtrics online interface, and the link to the survey was shared on Facebook groups dedicated to local communities (e.g., communities of particular towns or districts). Participants were informed that their participation is voluntary and they are free to withdraw from the survey at any stage without giving any reason. The mean time of filling out the questionnaire was about 9 min. The participants filled in the items in the above-described order—first the questions about the feeling of threat, and next the questionnaire on conspiracy beliefs and assessments of emotions. In the last block, the participants were asked if either they or their relatives suffered from COVID-19 disease. Then the respondents were indicating their gender, age, the size of the place they live in and their level of education. Lastly, respondents completed two items asking if they are full-time employees and if their remuneration payment is guaranteed during the pandemic.

## 3. Results

### 3.1. Correlates of the Feeling of Threat Caused by the COVID-19 Pandemic

We verified whether the variables measured in our study had a normal distribution with the Kolmogorov-Smirnov test—none of the variables had normal distribution (*p*(K-S) < 0.01), therefore we decided to use non-parametric correlation analysis—Spearman’s rho. We checked what factors correlate with the feeling of threat caused by the COVID-19 pandemic. All the emotional categories correlated positively with the feeling of being threatened, with the values of correlations differing from a medium for automatic homeostatic emotions (r = 0.573; *p* < 0.001) and sadness-like reflective emotions not related to Self (r = 0.498; *p* < 0.001) to low for automatic aversive emotions (r = 0.304; *p* < 0.001), reflective emotions related to Self standards (r = 0.247; *p* < 0.001) and contempt-like reflective emotions not related to Self (r = 0.111; *p* = 0.01). We did not find a significant correlation between age and feeling of threat. The belief in conspiracy theories (or any of its factors) also did not correlate with the feeling of threat, therefore we decided to not include this factor in further analyses. Descriptive statistics for the variables and values of the correlations are presented in Table 1.

In further analyses, we checked whether other factors can influence the general feeling of being threatened. The distributions of the feeling of threat in groups divided by gender, place of living, education or working in a full-time job did not fulfil normality criteria (*p*(K-S) < 0.001), therefore we used the *U* Mann-Whitney non-parametric test. We found that women are feeling more threatened by the coronavirus pandemic (*M* = 2.82; *SD* = 0.96) than men (*M* = 2.34; *SD* = 0.97; *U* = 88,553.50; *p* < 0.001). We did not find any other factors (place of living, education or working in a full-time job) to significantly influence the feeling of threat.

### 3.2. Predictors of the Feeling of Threat

All the variables that correlated with the feeling of threat (see Table 1), as well as gender as a dichotomic factor, were put in a linear regression model (Model 1). Apart from gender, all the variables were numeric. After excluding the non-significant predictors from the model one by one only two predictors remained significant, namely automatic homeostatic emotions (*β* = 0.643; *t* = 15.98; *p* < 0.001) and reflective emotions related to Self standards (*β* = −0.116; *t* = 2.89; *p* = 0.004), *F*(2, 568) = 150.367, *p* < 0.001, *R*^2^ = 0.346. This model could be presented in the form of an equation as (unstandardized Betas are used in the equation) *y* = 0.073*x*_1_ + (−0.016*x*_2_) + 5.08, where *y* = anxiety caused by the virus, *x*_1_ = automatic homeostatic emotions and *x*_2_ = reflective emotions related to Self standards.

### 3.3. Exploratory Analyses—Predictors of the Feeling of Threat in Different Age Groups

Based on the exploration of the sample regarding the frequency of participants of different ages, we selected two age groups: (1) young adults, ages ranging from 18 to 34 years old (*N* = 418; *M* = 24.45; *SD* = 4.11) and (2) older adults, age from 35 to 74 years old (*N* = 157; *M* = 42.16; *SD* = 7.02). We tested the regression models with variables, that were in significant relationship to the feeling of threat caused by the pandemic. We found, that in the group of young adults only automatic homeostatic emotions were a significant predictor of the feeling of threat (*M* = 49.26; *SD* = 25.73; *β* = 0.569; *t* = 14.13; *p* < 0.001), *F*(1, 416) = 199.523, *p* < 0.001, *R*^2^ = 0.324. The model could be presented as an equation, *y* = 0.062*x* + 4.951, where *y* = feeling of being threatened by the virus and *x* = automatic homeostatic emotions. In the group of older adults three emotional factors were predictors of the feeling of threat, two of them being risk factors, namely automatic homeostatic emotions (*M* = 46.98; *SD* = 27.05; *β* = 0.506; *t* = 4.03; *p* < 0.001) and sadness-like reflective emotions not related to Self (*M* = 47.20; *SD* = 30.61; *β* = 0.287; *t* = 2.45; *p* = 0.015), while one was a protective factor, namely reflective emotions related to Self standards (*M* = 33.38; *SD* = 24.15; *β* = −0.214; *t* = 2.80; *p* = 0.006), *F*(3, 153) = 39.45, *p* < 0.001, *R*^2^ = 0.436. The model could be presented in the form of an equation as *y* = 0.061*x*_1_ + 0.031*x_2_ +* (−0.029*x*_3_) + 4.935, where *y* = feeling of being threatened by the virus, *x*_1_ = automatic homeostatic emotions, *x*_2_ = sadness-like reflective emotions not related to Self and *x*_3_ = reflective emotions related to Self standards. All the final regression models (for all participants and different age groups) have been presented in Table 2.

### 3.4. Post-Hoc Statistical Power Analyses

Post-hoc verification of the statistical power of the study using G-Power 3.1. software [61] has revealed very high statistical power of correlations, ranging from 0.73 for reflective emotions not related to Self to 0.99 for automatic homeostatic emotions. It has also revealed a very high statistical power of 0.99 for the gender differences, as well as all final regression models. The noncentrality parameter *λ* for regression models ranged from 123.36 to 307.46.

## 4. Discussion

In the confirmatory part of our analyses, we found that all emotional factors correlated positively with the feeling of threat caused by the novel coronavirus. It is important to note, that all the emotions were negative, thus we can see that generally experiencing negative emotions is correlated with the feeling of being threatened—as it appeared in previous studies [11,18,19,21,51]. Nevertheless, we found that only two emotional categories were significant predictors of the feeling of threat caused by the pandemic. Automatic homeostatic emotions were a really strong predictor, which is a result that could have been expected. Automatic homeostatic emotions are basic feelings, related to keeping homeostasis in one’s body, and as COVID-19, being a contagious disease, is a serious threat to homeostasis, the accumulation of emotions from this cluster could easily amplify the anxiety. Interestingly, we found reflective emotions related to Self standards to be protective against the feeling of threat, despite the fact that they were still negative emotions. Experiencing reflective emotions, especially in such a difficult situation, may be an indicator of a well-developed pattern of affective processing [26], which may be the reason why we observe these emotions as being protective against a strong feeling of threat—the structure of emotional processing itself may be protective, allowing for an adequate appraisal of a threatening situation. This corresponds with results showing the influence of emotional intelligence as a trait, one of the main components of the structure of emotional processing, to moderate the feelings experienced during the pandemic [19].

When we divided participants into two groups, based on their age, we found that reflective emotions related to Self standards were protective against a strong feeling of threat only in the group of older participants. Also, reflective emotions not related to Self standards, but sadness-like, turned out to be a risk factor. Automatic homeostatic emotions, these basic emotional states related to physical well-being, were a risk factor in both age groups, and the only significant predictor of anxiety in the younger group. These results show a difference in emotional functioning in the face of the pandemic between people of different ages. As older people could be, in general, emotionally well-developed, they may experience more emotional states that are based on social constructs and need a cognitive appraisal. This may provoke them to generally employ the reflective, systematic brain in processing the information regarding the pandemic, thus reducing their anxiety level. This result of reflective emotions being protective for older adults confirms our previous findings [21], as well as findings from other studies showing different factors influencing COVID-related anxiety in different age groups [18,44]. Including reflective emotions in the span of affective processing means, that also positive reflective emotions would influence one’s thinking, and emotions such as compassion and hope, which also could support emotional well-being [42]. Young people on the other hand, who did not develop this kind of protective mechanism, seem to be overwhelmed by basic emotions, that cause anxiety and a strong feeling of threat.

We can conclude that young adults during the outburst of the pandemic were mostly focused on the immediate, direct influence of the situation, which reinforced the importance of automatic emotions related to homeostasis in their processing. Emotions such as helplessness, frustration or breakdown could be a direct response to lockdown, unsure job situation and danger of infection. For the emotional processing of older adults reflective emotions held much more importance (as they have a significant cognitive component) [62], which could be explained by perceiving the situation in a wider context provided by experience [63]. The context could also be supported by stronger ties to family and local communities [64,65], which could evoke negative emotions related to self-standards in relation to actions done by other people (e.g., *embarrassment*). Also, a much more pronounced realization of one’s goals in developed adulthood could be the cause of certain reflective emotions occurring, when achieving those goals seem to fade away in the face of the pandemic (this might have been visible in studies concerning the scientists during a pandemic—older principal investigators had significantly lower stress levels concerning acquiring goals of the project than younger ones) [66]. To sum up, the diversity of factors taken into consideration during constructing the emotional response to a novel situation supports a more comprehensive cognitive appraisal of emotional arousal itself, and in consequence, including the reflective, complex emotions in the span of feelings [26,67].

The results support the concept of automatic and reflective emotions being separate concepts, evoked by different mechanisms [26]. Origin of emotions is still a rather new concept, explored mostly in relation to cognitive processes [68] or decision-making [69,70], there are however almost no studies that show how the dimension of origin differentiates the feelings occurring naturally, not in experimental situations. Our study not only shows that there are differences in experiencing differently originated feelings in accordance with the proposed theoretical framework, but also how important identifying differently originated effects may be. It has to be mentioned here that the proposed group of reflective emotions not related to Self have not provided a reliable measure of the concept we proposed, with an appraisal of, for example, *sadness*, differing significantly from *envy* and *contempt.* This group of emotions, depending on external stimuli, may be highly dependent on the specificity of the situation, which (as we can see in the descriptive statistics) did not evoke strong *envy* or *contempt* in our participants.

We hope that the protective role of reflective emotions related to Self may be used in practice in psychotherapy, crisis intervention and public communication. Providing a wider perspective of a certain situation and, especially, putting it in a context including close ones and/or family members, could shift the focus from oneself and direct danger to body and health, to caring about more intangible ideas and caring about other people. Such reevaluation can lead to emotional growth, compassion and finding sense in a new situation [43].

As we expected, women were feeling more threatened by the coronavirus pandemic than men. This lies in line with many of the previous studies [9,14,19,51]. Women’s bigger vulnerability to anxiety during the pandemic can be caused by socialization processes and specific expectations of how women and men ought to express and handle anxiety and stress. Another explanation of this effect could be biological and evolutionary differences between men and women in vulnerability to anxiety—women, being more vulnerable from the evolutionary viewpoint in general, tend to be more anxious, to protect from potentially dangerous situations [71]. It has to be acknowledged that the participants in the study were mostly females, which means that the measurement for them was much more reliable than for males. This has to be taken into consideration when interpreting the results regarding gender, this may also be one of the reasons why gender is not a significant predictor of the feeling of threat in the regression models.

We did not find conspiracy beliefs to influence the feeling of threat caused by COVID-19 in the early days of the pandemic. It has to be mentioned that many of the conspiracy theories that emerged around the pandemic were related to decisions of governments and vaccines, topics that were present in the media months after the first wave of infections [72,73]. This also seems to be in line with similar studies conducted on Polish samples [74]. It appears that the measure of general belief in conspiracies was not relatable to the content of the new conspiracy theories emerging during the pandemic, therefore it did not bring reliable insight into the actual relation between conspiratorial thinking and the feeling of threat. On the other hand, in the comprehensive regression model, we also did not observe the influence of other factors, such as economic situation or education, on the feeling of threat caused by the virus. This could mean that in the research sample, the diversity of emotional experiences was the main factor influencing the feeling of threat. This claim however requires more investigation, regarding the exact stimuli evoking both automatic and reflective emotions (such as news reported by media, but also changes in a job situation, as well as private and family life), but also traits moderating the emotional processing [44]. The possibility to compare our results with other studies conducted on Polish participants is also limited due to different translations of the GCB Scale being used in research [60,74].

The specificity of the participants could be the most important limitation of the study. First, the participants were mostly women, which limits the generalization of the results. Secondly, the people that fulfilled out the questionnaire were social media users, interested in the topic of the novel coronavirus pandemic to such an extent, to spend a few minutes answering the questions with no remuneration provided. We surmise that people who are generally not interested in this topic would not even click the link to the questionnaire. This means that the relations between factors measured in this study, especially the ones between emotions and the feeling of threat, could have a different shape or may even not appear at all in groups of people not involved in the study. It could be especially different for some health professionals (e.g., doctors, nurses), who have a very specific experience with a pandemic, as they saw its impact every day at work. Having this direct contact with the pandemic can definitely have an influence on all of our measured variables; therefore, our findings should not be generalized to such specific groups.

It is also important to note, that for the use of the study, we developed a scale to measure the feeling of threat caused by the pandemic. Simultaneously in different countries researchers were also developing similar scales, differing in complexity and factors that were measured by them. One of the great examples could be a scale developed strictly for the sake of measuring anxiety related to the novel coronavirus pandemic [59]. The usage of different scales could be a limitation when comparing results from different studies and it is something that should be remembered when the results are interpreted. It also should be noted, that regardless of the fact that we measured negative emotions and the feeling of threat with separate scales, the constructs measured by both scales may overlap to some extent. This means that it is difficult to make conclusions about the influence of particular emotions on anxiety related to coronavirus, only the influence of certain groups of emotions could have been reliably analysed. It is also worth noting that we did not control for individual mental health issues (such as adjustment disorders, anxiety, depression etc.), which also could limit the generalization of the results.

## 5. Conclusions

The most important result of this study is the finding that reflective emotions can be a protective factor against a strong feeling of threat caused by the novel coronavirus spread. These results seem to bring a clear message for anxiety interventions in the face of the pandemic. Promoting reflective thinking and reworking emotions in complex, social contexts can lower stress and anxiety, maybe promoting emotional growth from the crisis situation. The influence of positive emotions on the feeling of threat needs to be further explored, but it seems that also among positive emotions the reflective ones could play an important role as a protective factor in the face of threat.

## Figures and Tables

**Table 1:** Correlates of the feeling of threat caused by the COVID-19 pandemic (*N* = 575). The table presents Min–Max values, Means and Standard Deviations for all the variables, as well as values of Pearson’s r correlations and statistical significance. Sadness-like and contempt-like emotions are reflective emotions not related to Self standards.

Variable			Feeling of Threat	
	*Min–Max*	*M (SD)*	*r*	*p*
Feeling of threat	1–7	2.70 (0.98)	-	-
Automatic homeostatic emotions	1–100	48.64 (26.09)	0.573	<0.001
Automatic aversive emotions	1–100	25.24 (23.77)	0.304	<0.001
Reflective emotions related to Self standards	1–100	34.37 (22.25)	0.247	<0.001
Sadness-like emotions	1–100	50.28 (29.75)	0.498	<0.001
Contempt-like emotions	1–100	19.72 (24.88)	0.111	0.01
Belief in conspiracy theories	1–5	2.47 (0.90)	0.06	0.147
Age of participant	18–74	29.38 (9.34)	0.052	0.210

**Table 2:** Regression models differ with the age of participants and statistically significant predictors of the feeling of threat caused by the pandemic. Emotion categories: auto homeo = automatic homeostatic emotions; refl Self = reflective emotions related to Self; refl sadness = sadness-like reflective emotions not related to Self.

Model	Age	*N*	*F*	*R* ^2^	*p(F)*	Predictor	*β*	*p*(*β*)
Model 1.	18–74	571	150.367	0.346	<0.001			
						auto homeo	0.646	<0.001
						refl Self	−0.118	0.004
Model 2.	18–34	418	199.523	0.324	<0.001			
						auto homeo	0.569	<0.001
Model 3.	35–74	157	39.45	0.436	<0.001			
						auto homeo	0.506	<0.001
						refl sadness	0.287	0.015
						refl Self	−0.214	0.006

## Data Availability

All of the data obtained in the experiment are publicly available in the Figshare repository: https://doi.org/10.6084/m9.figshare.22059416.v2 accessed on 17 March 2023.

## Supplementary Materials

The following supporting information can be downloaded at: https://www.mdpi.com/article/10.3390/ijerph20075231/s1, Table S1: Materials used in the online survey, with subscales, number of items and results of validation analyses (*N* = 575).
