# Supplementary material for: Predictors of Feeling of Threat Caused by COVID-19 Pandemic, the Distinctive Effects of Automatic vs. Reflective Emotions
Source: Int J Environ Res Public Health. 2023 Mar 23;20(7):5231. doi: 10.3390/ijerph20075231 (PMC10094237; doi:10.3390/ijerph20075231)
Supplement: Supplementary file 1 [file ijerph-20-05231-s001.zip › ijerph-2241374-supplementary.pdf]

Supplementary Materials

Table S1. Materials used in the online survey, with subscales, number of items and results of validation analyses (N = 575).
The questionnaire marked with "-" was not treated as an individual scale.

Questionnaire	Scale (a-priori set)	Subscale (exploratory found)	Number of items	Validation (Cronbach's α)
Feeling of threat caused by COVID-19 pandemic			3	0.77
Generic Conspiracist Beliefs Scale (Brotherton et al., 2013)			15	0.93
	Government Malfeasance		3	0.81
	Malevolent Global		3	0.87
	Extraterrestrial Cover- up		3	0.85
	Personal Wellbeing		3	0.78
	Control of Information		3	0.73
Negative Emotions			-	-
	Automatic homeostatic		5	0.87
	Automatic heterostatic		5	0.86
	Reflective related to Self standards		5	0.78
	Reflective not related to Self standards		5	0.75
		Sadness-like	3	0.86
		Contempt-like	2	$r = 0.55$; $p < 0.001$
